# Natural and Artificial Dynamics in Graphs: Concept, Progress, and Future

**DOI:** 10.3389/fdata.2022.1062637

**Published:** 2022-12-02

**Authors:** Dongqi Fu, Jingrui He

**Affiliations:** ^1^Department of Computer Science, University of Illinois at Urbana-Champaign, Champaign, IL, United States; ^2^School of Information Sciences, University of Illinois at Urbana-Champaign, Champaign, IL, United States

**Keywords:** graph mining, graph representations, graph neural networks, natural dynamics, artificial dynamics

## Abstract

Graph structures have attracted much research attention for carrying complex relational information. Based on graphs, many algorithms and tools are proposed and developed for dealing with real-world tasks such as recommendation, fraud detection, molecule design, etc. In this paper, we first discuss three topics of graph research, i.e., graph mining, graph representations, and graph neural networks (GNNs). Then, we introduce the definitions of *natural dynamics* and *artificial dynamics* in graphs, and the related works of natural and artificial dynamics about how they boost the aforementioned graph research topics, where we also discuss the current limitation and future opportunities.

## 1. Introduction

In the era of big data, the relationship between entities becomes much more complex than ever before. As a kind of relational data structure, graph (or network) attract much research attention for dealing with this unprecedented phenomenon. To be specific, many graph-based algorithms and tools are proposed, such as DeepWalk (Perozzi et al., 2014), LINE (Tang et al., 2015), node2vec (Grover and Leskovec, 2016), GCN (Kipf and Welling, 2017), GraphSAGE (Hamilton et al., 2017), GAT (Velickovic et al., 2018), etc. Correspondingly, many challenges of real-world applications get addressed to some extent, such as recommendation (Fan et al., 2019), fraud detection (Wang et al., 2019), and molecule design (Liu et al., 2018), to name a few.

To investigate graph-based research and relevant problems and applications systematically, at least^1^ three aspects will be discussed, i.e., graph mining, graph representations, and graph neural networks (GNNs). Their dependency is convoluted, the reason why we aim to disentangle it is that we can discuss the current efforts from natural and artificial dynamics studies (which are improving the graph algorithms and tools performance) in a fine-grained view, such that we can envision detailed future research opportunities. As for **natural dynamics in graphs**, we use this term to illustrate that the input graphs themselves are evolving, i.e., the topology structures, the node-level, edge-level, and (sub)graph-level features and labels are dependent on time (Aggarwal and Subbian, 2014; Kazemi et al., 2020). As for **artificial dynamics in graphs**, we use this term to describe that end-users *change (e.g., filter, mask, drop, or augment) the existing* or *construct (i.e., from scratch) the non-existing* graph-related elements (e.g., graph topology, graph stream, node/graph attributes/labels, GNN gradients, GNN layer connections, etc.) to realize the certain performance upgrade [e.g., computation efficiency (Fu et al., 2020e), model explanation (Fu and He, 2021b), decision accuracy (Zheng et al., 2022), etc.]. To the best of our knowledge, the first relevant act of conceiving artificial dynamics in graphs appeared in Kamvar et al. (2003), where “artificial jump” is proposed to adjust the graph topology for PageRank realizing the personal ranking function on structured data (Kamvar et al., 2003), i.e., a random surfer would follow an originally non-existing but newly-added highway to jump to a personally-selected node with a predefined teleportation probability.

With the above introduction of graph research terminology and dynamics category, in this paper, we are ready to introduce some related works on investigating natural and artificial dynamics in graph mining, graph representations, and graph neural networks, and then discuss future research opportunities. To be specific, this survey is organized as follows. The definition and relation introduction for graph mining tasks, graph representations, and graph neural networks are discussed in Section 2. Then, in Section 3, we discuss the formal definition followed by concrete research works for *natural dynamics, artificial dynamics*, and *natural + artificial dynamics* in graphs. Finally, in Section 4, we conclude the paper with sharing some research future directions.

## 2. Relations among graph mining, graph representations, and graph neural networks

To pave the way for investigating the natural and artificial dynamics in graphs, we first introduce graph research topics (i.e., graph mining, graph representations, and graph neural networks) and their relationships in this section. Then, in the next section, we can target each topic and see how natural dynamics and artificial dynamics contribute to them.

**Figure 1:**
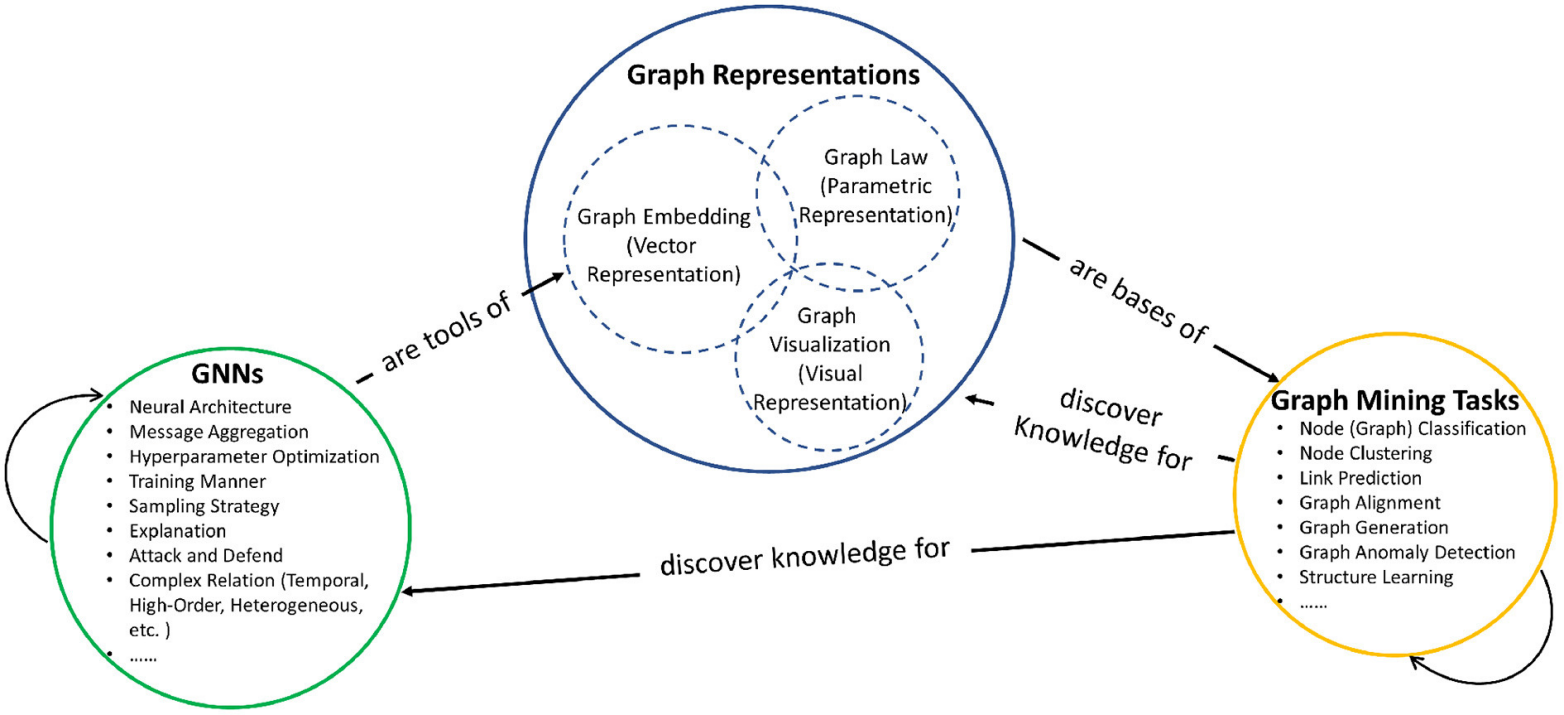
Relationships among graph mining, graph representations, and graph neural networks.

In general, the relationships between graph mining, graph representations, and graph neural networks can be illustrated as shown in Figure 1. (1) Graph mining aims to extract interesting (e.g., non-trivial, implicit, previously unknown, and potentially useful) knowledge from graph data, and graph mining consists of numerous specific tasks, such like node classification (Kipf and Welling, 2017) is aiming to classify the node category based on its features, and node clustering (Shi and Malik, 2000; Andersen et al., 2006) is aiming to partition the entire graph into disjoint or overlapped clusters (i.e., subgraphs) based on end-users' objectives (e.g., conductance, betweeness, etc.). For example, clustering can discover knowledge to help GNN implementations, and Cluster-GCN (Chiang et al., 2019) is proposed to sample nodes in a topology-preserved clustering, which could entitle vanilla GCN (Kipf and Welling, 2017) the fast computation to deal with large-scale graph datasets. (2) Graph representations are the bases of graph mining, which projects graphs into a proper space such that graph mining can do various task-specific computations. To the best of our knowledge, graph representations consists of three components. First, graph embedding represents graphs with affinity matrices like Laplacian matrix and hidden feature representation matrix, on which many mining tasks rely, such as node classification (Kipf and Welling, 2017); Second, graph law represents graphs with several parameters which describe the statistical property of graphs such as node degree distribution and edge connection probability, which could help mining tasks like graph generation (Leskovec and Faloutsos, 2007) and link prediction (Wang et al., 2021b); Third, graph visualization provides the visual representations and can serve for the domain-specific knowledge interpretation (Bach et al., 2015; Yang Y. et al., 2020). Within graph representations, graph embedding, graph law, and graph visualization can contribute to each other, and detailed overlapping works are discussed in the following sections. (3) Graph neural network (GNN) is an effective tool for extracting meaningful graph embedding vectors (or matrices) by combining deep learning theory and graph theory (Wu et al., 2021). Graph neural networks are composed of a family of many specific models with different research concerns like neural architecture (Chen M. et al., 2020) and message passing aggregation design (Klicpera et al., 2019), the detailed related works are also discussed in the following sections.

### 2.1. Graph mining

Graph mining interacts with real-world problems by discovering knowledge for many applications. Based on structured data, graph mining consists of numerous specific tasks. For example,

- Node (and graph) classification (Kipf and Welling, 2017; Zhang et al., 2018; Jing et al., 2021): Nodes sharing similar features should be classified into the same category.
- Node clustering (or graph partitioning) (Shi and Malik, 2000; Ng et al., 2001; Andersen et al., 2006; Spielman and Teng, 2013): Individual nodes are clustered for optimizing certain metrics such as inter-cluster distance, intra-cluster density, etc.
- Link prediction (Dunlavy et al., 2011; Zhang and Chen, 2018; Kumar et al., 2019): The probability is estimated that whether two nodes should be connected based on evidence like node structural and attribute similarity.
- Graph generation (Leskovec and Faloutsos, 2007; Bojchevski et al., 2018; You et al., 2018; Zhou et al., 2019, 2020): Model the distribution of a batch of observed graphs and then generate new graphs.
- Subgraph matching (Tong et al., 2007; Zhang et al., 2009; Du et al., 2017; Liu et al., 2021): Check whether a query graph (usually the smaller one) can be matched in a data graph (usually the larger one) approximately or exactly.
- Graph anomaly detection (Akoglu et al., 2015; Yu et al., 2018; Zheng et al., 2019): Identify whether the graph has abnormal entities like nodes, edges, subgraphs, etc.
- Graph alignment (Zhang and Tong, 2016; Yan et al., 2021a,b; Zhou et al., 2021): Retrieve similar structures (e.g., nodes, edges, and subgraphs) across graphs.
- many more …

Those tasks can be directly adapted to solve many high-impact problems in real-world settings. For example, through learning the graph distribution and adding specific domain knowledge constraints, graph generators could contribute to molecule generation and drug discovery (Liu M. et al., 2022; Luo and Ji, 2022); With modeling picture pixels as nodes, graph partitioning algorithms could achieve effective image segmentation at scale (Bianchi et al., 2020); By modeling the information dissemination graph over news articles, readers, and publishers (Nguyen et al., 2020) or modeling the suspicious articles into word graphs (Fu et al., 2022a), node and graph classification tasks can help detect fake news in the real world.

### 2.2. Graph representations

For accomplishing various graph mining tasks, graph representations are indispensable for providing the bases for task-specific computations. To the best of our knowledge, graph representations can be roughly categorized into three aspects, (1) graph embedding (i.e., vector representation), (2) graph law (i.e., parametric representation), and (3) graph visualization (i.e., visual representation).

#### 2.2.1. Graph embedding (vector representation)

**First, graph representations can be in the form of embedding matrices**, i.e., the graph topological information and attributes are encoded into a matrix (or matrices). The most common form can be the Laplacian matrix, which is the combination of the graph adjacency matrix and degree matrix. Recently, the graph embedding (or graph representation learning) area attracts many research interests, along with numerous graph embedding methods proposed for extracting the node (or graph) hidden representation vectors from the input affinity matrices, like DeepWalk (Perozzi et al., 2014), LINE (Tang et al., 2015), and node2vec (Grover and Leskovec, 2016). They^2^ share the general principle to extract node representation vectors, which means a node could reflect (e.g., predict, be proximate to, etc.) its sampled neighbors in the embedding space, e.g., Skip-gram in Perozzi et al. (2014) and Grover and Leskovec (2016) and order-based proximity in Tang et al. (2015). With the different angles of viewing graph topology and node features, some derivatives are proposed, such as metapath2vec (Dong et al., 2017) for heterogeneous networks, graph2vec (Narayanan et al., 2017) for the graph-level embeddings, and tdGraphEmbd (Beladev et al., 2020) for temporal graph-level embeddings.

All graph embedding works mentioned above are unsupervised, which means the guidance (or constraints, regularizers) during the learning process are totally from the input graph structure and features, such that the encoded vectors within specific dimensions are actually reflecting the graph itself information. Hence, by involving extra domain knowledge (i.e., labels and task-specific loss functions), graph embedding vectors can serve real-world applications. For example, with user-item interaction history records and user anomaly labels, graph embedding techniques can be leveraged for predicting the user-interested merchandise and user's behavior in the future (Kumar et al., 2019); By involving additional labels, graph embedding vectors can be used to generate small molecule graphs through an encoder-decoder framework (Jin et al., 2018; Simonovsky and Komodakis, 2018); Also, with delicately designed query questions and temporal knowledge graphs, graph embedding techniques can be used to help answer open-world questions (Saxena et al., 2021; Shang et al., 2022).

#### 2.2.2. Graph law (parametric representation)

**Second, graphs can also be represented by several parameters**. A simple but common example is Erdős-Rényi random graph, i.e., *G*(*n, p*) or *G*(*n, m*) (Drobyshevskiy and Turdakov, 2020). To be specific, in *G*(*n, p*), the possibility of establishing a single edge among *n* nodes is independent of each other and valued by a constant parameter *p*; while in *G*(*n, m*), an *n*-node and *m*-edge graph is chosen evenly from all possible *n*-node and *m*-edge graph collections. In addition to the number of nodes, the number of edges, and the edge probability, many common parameters are well-studied for representing or modeling graphs, such as degree distribution, effective diameter, clustering coefficient, and many more (Chakrabarti and Faloutsos, 2006; Drobyshevskiy and Turdakov, 2020).

Representing graphs by graph laws can be summarized into the following steps: (1) determine the parameter (or formula of several parameters) to represent the graphs, (2) fit the value of parameters based on the graph structures and features through statistical procedures. For example, Leskovec et al. (2005) discover the densification law over evolving graphs in the macroscopic view, which is expressed as *e*(*t*) ∝ *n*(*t*)^α^, and *e*(*t*) denotes the number of edges at time *t*, *n*(*t*) denotes the number of nodes at time *t*, and α ∈ [1, 2] is an exponent parameter representing the density degree. And they use the empirical observation of real-world graphs to fit the value of α. Targeting the microscopic view, Leskovec et al. (2008) discover other graph laws. Different from the macroscopic view, they view temporal graphs in a three-fold process, i.e., node arrival (determining how many nodes will be added), edge initiation (how many edges will be added), and edge destination (where are the added edges), where they ignore the deletion of nodes and edges. Then, they assign variables and corresponding equations (i.e., models) to parameterize these three processes and use MLE (i.e., maximum likelihood estimation) to settle the model and scalar parameters based on real-world graph observation. As an instance, the edge destination (i.e., the probability for node *u* connecting node *v*) is modeled as *last*^τ^ other than *deg*^τ^ for the LinkedIn network through MLE, where *deg*^τ^ means the connection probability is proportional to node *v*'s current degree $d_{t}{\left(v\right)}^{\tau }$. And *last*^τ^ means the probability is proportional to node *v*'s age since its last interaction $\delta_{t}{\left(v\right)}^{\tau }$, where τ is the parameter to be fit.

Discovering graph laws and fitting law corresponding parameters can also serve many graph mining tasks and real-world applications. For example, after a graph law is discovered, the follow-up action is to propose the corresponding graph generative model to test whether there exists a realizable graph generator could generate graphs while preserving the discovered law in terms of graph properties (Leskovec et al., 2005, 2008; Park and Kim, 2018; Zang et al., 2018; Do et al., 2020; Kook et al., 2020; Zeno et al., 2020). Recently, the triadic closure law on temporal graphs (i.e., two nodes that share a common neighbor directly tend to connect) has been discovered to contribute to the dynamic link prediction task (Wang et al., 2021b). For the questions in social network analysis, e.g., “What is Twitter?”, Kwak et al. (2010) give the statistical answer in the form of parametric representation. For pre-training the language model, the values of the weighted word co-occurrence matrix (i.e., adjacency matrix) are necessary and highly depend on the parameters following the power law, e.g., in GloVe (Pennington et al., 2014), *X*_*ij*_ denotes the number of times that word *j* occurs in the context of word *i*, and it follows $X_{ij}=\frac{k}{{\left(r_{ij}\right)}^{\alpha }}$, where *r*_*ij*_ denotes the frequency rank of the word pair *i* and *j* in the whole corpus, and *k* and α are constant parameters.

#### 2.2.3. Graph visualization (visual representation)

**Third, graph visualization provides visual representation by plotting the graph directly**, which is more straightforward than graph embedding and graph law to some extent. Hence, one of the research goals in graph visualization is finding the appropriate layout for the complex networked data. To name a few: most graphs (e.g., a five-node complete graph) could not be plotted on the plane without edge crossings, then Chen K. et al. (2020) give the solution about how to use a 3D torus to represent the graph and then flatten the torus onto the 2D plane with aesthetics and representation accuracy preserved; Also, in Nobre et al. (2020), authors evaluate which layouts (e.g., node-link diagram or matrix) are suitable for representing attributed graphs for different graph mining tasks; Through crowd-sourced experiments, Yang Y. et al. (2020) study the tactile representation of graphs for low-vision people and discuss which one (e.g., text, matrix, or node-link diagram) could help them to understand the graph topology; When the graph is large (e.g., hundreds of thousands of nodes), it is hard to represent the internal structure, and Nassar et al. (2020) design the high-order view of graphs (i.e., construct *k*-clique weighted adjacency matrix) and then use t-SNE to get the two-dimensional coordinates from the weighted Laplacian matrix. Bringing time information to graph visualization started in the 1990s to deal with the scenario where the represented graph gets updated (Beck et al., 2014). The trend for visualizing dynamic (or temporal) graphs becomes popular, and different research goals emerge (Kerracher et al., 2014; Beck et al., 2017), like strengthening the domain-specific evolution for domain experts (Bach et al., 2015), showing the pandemic dissemination (Lacasa et al., 2008; Tsiotas and Magafas, 2020), explaining time-series data (e.g., response time to different questions) with graph visualization and graph law (Mira-Iglesias et al., 2019).

Plotting graphs into an appropriate layout is more challenging when it comes to complex evolving graphs. Hence, many dynamic graph visualization research works contribute their solutions from different angles. For example, for balancing the trade-off between temporal coherence and spatial coherence (i.e., preservation of structure at a certain timestamp), Leydesdorff and Schank (2008) use the multidimensional scaling (MDS) method. Inspired by that, Xu et al. (2013) design the dynamic multidimensional scaling (DMDS), and Rauber et al. (2016) design the dynamic t-SNE; In order to assign end-users the flexibility to view the different aspects of evolving graphs (e.g., time-level graph evolution or node-level temporal evolution), Bach et al. (2014) represent evolving graphs into user-rotating cubes; To highlight the temporal relation among graph snapshots, authors in Bach et al. (2016) propose Time Curves to visualize the temporal similarly between two consecutively observed adjacency matrices; In Lentz et al. (2012) and Pfitzner et al. (2012), researchers find that paths in temporal networks may invalidate the transitive assumption, which means the paths from node *a* to node *b* and from node *b* to node *c* may not imply a transitive path from node *a* via node *b* to node *c*. Inspired by this observation and to further analyze the actual length of paths in temporal graphs, Scholtes (2017) transfer this problem into investigating the order (i.e., *k*) of graphs. To be specific, the order *k* can be understood as the length of a path (i.e., *v*_*i*−*k*_ → … → *v*_*i*−1_ → *v*_*i*_) and can be modeled by the high-order Markov Chain [i.e., ℙ(*v*_*i*_|*v*_*i*−*k*_ → … → *v*_*i*−1_)]. And the order of temporal paths can be determined by thresholding the probability gain in the MLE model. A corresponding follow-up visualization work is proposed targeting the high-order temporal graphs (Perri and Scholtes, 2019), which first determines the order of a temporal network as discussed above, and then constructs intermediate supernodes for deriving the high-order temporal relationship between two nodes, finally plots this high-order temporal relationship into edges and adds them on a static graph layout.

### 2.3. Graph neural networks

To extract the hidden representation, graph neural network (GNN), as a powerful tool, provides a new idea different from the embedding methods like DeepWalk (Perozzi et al., 2014), LINE (Tang et al., 2015), and node2vec (Grover and Leskovec, 2016). One major difference between GNNs and those mentioned above is that GNNs could aggregate multi-hop node features to represent a node by stacking GNN layers. According to Xu K. et al. (2019), this mechanism is called information aggregation (or message-passing in some literature), which iteratively updates the representation vector of a node by aggregating the representation vectors from its neighbors. The general formula of GNNs can be expressed as follows.


(1) $$\begin{array}{l} a_{v}^{\left(k\right)}=AGGREGATE^{\left(k\right)}(\{h_{u}^{\left(k-1\right)}:u\in 𝒩(v)\}),\\ \;h_{v}^{\left(k\right)}=COMBINE^{\left(k\right)}(h_{v}^{\left(k-1\right)},a_{v}^{\left(k\right)}) \end{array}$$

where ${\text{h}}_{v}^{\left(k\right)}$ is the hidden representation vector of node *v* at the *k*-th iteration (i.e., *k*-th layer), and ${\text{a}}_{v}^{\left(k\right)}$ is the aggregation among hidden representation vectors of neighbors N(v) of node *v* from the last iteration (i.e., layer). For example, the graph convolutional neural network (GCN) (Kipf and Welling, 2017) can be written in the above formulation by integrating the *AGGREGATE* and *COMBINE* as follows.


(2) $$\begin{array}{l} {\text{h}}_{v}^{\left(k\right)}=ReLU\left({\text{W}}^{\left(k-1\right)}\cdot MEAN\left\{{\text{h}}_{u}^{\left(k-1\right)},\forall u\in N\left(v\right)\cup \left\{v\right\}\right\}\right) \end{array}$$

where **W**^(*k*−1)^ is a learnable weight matrix at the (*k*−1)-th layer, and the original equation of GCN is as follows.


(3) $$\begin{array}{l} {\text{H}}^{\left(k\right)}=ReLU\left(\hat{\text{A}}{\text{H}}^{\left(k-1\right)}{\text{W}}^{\left(k-1\right)}\right) \end{array}$$

where **Â** is the normalized adjacency matrix with self-loops, i.e., $\text{Â}={\tilde{\text{D}}}^{-\frac{1}{2}}\tilde{\text{A}}{\tilde{\text{D}}}^{-\frac{1}{2}}$, and $\tilde{\text{A}}=\text{A}+\text{I}$.

Graph neural network is a complicated computational framework that integrates the neural networks from deep learning and non-Euclidean constraints from graph theory. Therefore, GNN research consists of many specific facets from both ends. For example,

- Neural layer architecture design: Recurrent (Li et al., 2018; Hajiramezanali et al., 2019), Residual Connections (Chen M. et al., 2020; Zheng et al., 2022), etc.
- Message passing schema: Spectral convolution (Kipf and Welling, 2017), Spatial convolution (Velickovic et al., 2018), Simplification (Klicpera et al., 2019; Wu et al., 2019), etc.
- Training manner: Semi-supervised learning (Kipf and Welling, 2017), Self-supervised learning (Velickovic et al., 2019; You et al., 2020), etc.
- Sampling strategy: Noises-aware (Yang Z. et al., 2020), Efficiency and generalization (Hu S. et al., 2020), Fairness-preserving (Kang et al., 2022), etc.
- Model trustworthy: Attack and defend (Zhu et al., 2019; Zhang and Zitnik, 2020), Black-box explanation (Ying et al., 2019; Luo et al., 2020; Vu and Thai, 2020), etc.
- many more …

Until now, we have introduced three aspects of graph research shown in Figure 1. Targeting each aspect, research in natural and artificial dynamics could contribute to performance improvements. The detailed related works are discussed in the next section, where we start by defining the natural and artificial dynamics in graphs, and then investigate how natural and artificial dynamics help graph research enhancements in each specific aspect.

## 3. Natural and artificial dynamics in graphs

**Natural dynamics in graphs** means that the input graph (to graph mining, graph representations, and GNNs) has the naturally evolving part(s), such as the evolving World Wide Web. Formally speaking, the naturally evolving part means that the topological structures or node (edge, subgraph, or graph) features and labels depend on time. To be specific, the evolving graph structures can be represented either in

- (1) continuous time (Kazemi et al., 2020) or streaming (Aggarwal and Subbian, 2014): an evolving graph can be modeled by an initial state *G* with a set of timestamped events *O*, and each event can be node/edge addition/deletion; or
- (2) discrete time (Kazemi et al., 2020) or snapshots (Aggarwal and Subbian, 2014): an evolving graph can be modeled as a sequence of time-respecting snapshots *G*^(1)^, *G*^(2)^, …, *G*^(*T*)^, and each *G*^(*t*)^ has its own node set *V*^(*t*)^ and edge set *E*^(*t*)^.

For these two modelings, the corresponding time-dependent features and labels can be represented in a time-series or a sequence of matrices such as **X**^(1)^, **X**^(2)^, …, **X**^(*T*)^.

These two modeling methods have non-trivial complements. For example, continuous-time models rapid node/edge-level evolution, i.e., microscopic evolution (Leskovec et al., 2008), such as protein molecule interactions in a cell (Fu and He, 2021a); However, it could not represent the episodic and slowly-changing evolution patterns, which can be captured by discrete-time, i.e., macroscopic evolution (Leskovec et al., 2005), such like the periodical metabolic cycles in a cell (Fu and He, 2021a). Recently, different evolution patterns in a single graph are currently not jointly modeled for improving graph representation comprehensiveness, but some real-world evolving graphs naturally have both evolution patterns. For example, in Fu and He (2021a), each dynamic protein-protein interaction network has 36 continuous observations (i.e., 36 edge timestamps), every 12 observations compose a metabolic cycle (i.e., three snapshots), and each cycle reflects 25 mins in the real world. Inspired by this observation, a nascent work (Fu et al., 2022b) is recently proposed to jointly model different evolution patterns into the graph representation.

**Artificial dynamics in graphs** means that the graph research related elements (e.g., graph topology, graph stream, node/graph attributes/labels, GNNs gradients, and neural architectures, etc.) are deliberately re-designed by end-users for boosting the task performance in certain metrics. For the re-designing, end-users can *change (e.g., filter, mask, drop, or augment) the existing elements* or *construct (i.e., from scratch) non-existing elements* to improve the performance (e.g., decision accuracy, model robustness, and interpretation, etc.) than the original. To name a few, one example of artificial dynamics can be graph augmentation: DropEdge (Rong et al., 2020) is proposed to deal with the over-fitting of GNNs by randomly removing a certain amount of edges from the input graphs for each training epoch; DummyNode (Liu X. et al., 2022) is proposed to add a dummy node to the directed input graph, which connects all existing *n* nodes with 2*n* directed edges. The dummy node serves as a highway to extend the information aggregation in GNNs and contribute to capturing the global graph information, such that the graph classification accuracy by GNNs can be enhanced. In addition to the graph augmentation, other specific examples of artificial dynamics can be filtering unimportant coming sub-structures to save computations (Fu et al., 2020e), adding residual connections among GNNs layers to address vanishing gradients (Zheng et al., 2022), and perturbing the GNNs gradients for privacy protection (Yang et al., 2021).

As mentioned above, on the one hand, considering the natural dynamics could leverage temporal dependency to contribute to graph research in terms of but not limited to, fast computation (e.g., tracking from the past instead of computing from scratch), causality reasoning (e.g., previous states cause the current state), comprehensive decision (e.g., prediction based on historical behaviors); On the other hand, studying artificial dynamics could help a wide range of targets, such as machine learning effectiveness (e.g., robustness, de-overfitting, de-oversmoothing).

Investigating natural dynamics and investigating artificial dynamics not only have shared merits but also have exclusive advantages. For example, how to manipulate evolving graphs is still an opening question for many downstream task improvements. Thus, a spontaneous research question is to ask whether natural dynamics can be integrated with artificial dynamics, which aims to keep the shared merits and bring exclusive advantages to synergy complementation. Definitely, some pioneering works have been proposed to touch this area. To introduce them, throughout the paper, we use **natural + artificial dynamics** to denote the integrated investigation of natural dynamics and artificial dynamics in graph-related research and then present related works in this category.

Starting from the following subsections, we are ready to introduce recent related works about natural, artificial, and natural + artificial dynamics research in graph mining, graph representations, and GNNs, respectively.

### 3.1. Dynamics in graph mining

Graph mining is a general term that consists of various specific mining tasks on graphs. Classic graph mining tasks consist of node clustering (or graph partitioning), node/graph classification, and link prediction. Also, motivated by real world application scenarios, novel graph mining tasks are being proposed for research, such as graph generation, graph alignment, and many more. Facing various graph mining tasks, we discuss several graph mining tasks here and then introduce the corresponding related works of natural dynamics, artificial dynamics, and natural + artificial dynamics in each discussed task.

#### 3.1.1. Natural dynamics in graph mining

**Link prediction**. The core of the link prediction task is to decide whether there should be a link between two entities in the graph. This graph mining task can directly serve the recommender system by modeling the user and items as nodes in their interaction graphs. The evidence to decide whether two nodes should be linked can be the current heuristics like node embedding similarity (Zhang and Chen, 2018; Zhu et al., 2021), and also the historical behaviors of entities can be added for a more comprehensive decision. For example, JODIE (Kumar et al., 2019) is a link prediction model proposed based on user-item temporal interaction bipartite graph, where a user-item interaction is modeled as (*u, i, t*, **f**) that means an interaction happens between user *u* and item *i* at time *t*, and **f** is the input feature vector of that interaction. Given a user (or an item) has a sequence of historical interactions (i.e., a user interacts with different items at different timestamps), JODIE (Kumar et al., 2019) applies two mutually-recursive RNN structures (i.e., *RNN*_*U*_ and *RNN*_*I*_) to update the embedding for users and items as follows.


(4) $$\begin{array}{l} u(t)=\sigma (W_{1}^{u}\;u(t^{-})+W_{2}^{u}\;i(t^{-})+W_{3}^{u}\;f+W_{4}^{u}\;{\text{Δ}}_{u}),\\ \text{ embedding unit of }RNN_{U}\\ \;i(t)=\sigma (W_{1}^{i}\;i(t^{-})+W_{2}^{i}\;u(t^{-})+W_{3}^{i}\;f+W_{4}^{i}\;{\text{Δ}}_{i}),\\ \text{ embedding unit of }RNN_{I} \end{array}$$

where ${\text{W}}_{1}^{u}$, ${\text{W}}_{2}^{u}$, ${\text{W}}_{3}^{u}$, and ${\text{W}}_{4}^{u}$ are four parameters of *RNN*_*U*_. And *RNN*_*U*_ and *RNN*_*I*_ share the same intuitive logic. Suppose user *u* interacts with item *i* at time *t* with the interaction feature **f**, then the above equation *RNN*_*U*_ updates the user embedding **u**(*t*) at time *t* by involving the latest historical user and item behavior, where Δ_*u*_ denotes the time elapsed since user *u*'s previous interaction with any item, **u**(*t*^−^) denotes the latest user embedding vector right before time *t*, and **i**(*t*^−^) denotes the latest item embedding vector right before time *t*. Therefore, in JODIE, each user (or item) can have a sequence of embedding vectors, which is called its trajectory. And the user and item embeddings can be updated iteratively to the future. The training loss is designed for whether the future user (or item) embedding vectors can be predicted.^3^ If the future embedding can be predicted [e.g., *u* connects *i* at *t*, and **i**(*t*) is predicted through **u**(*t*^−^) and **i**(*t*^−^)], then the user (or item) historical evolution pattern is supposed to be encoded. Thus, the trained model can be used to predict whether a user *u* interacts with an item *i* in the future.

**Graph alignment**. Compared with classic graph mining tasks, graph alignment is a relatively novel graph mining task, aiming to find paired (i.e., similar) nodes across two graphs. The input graphs can be attributed (e.g., heterogeneous information networks or knowledge graphs), and the proximity to decide whether two nodes from two different graphs are paired or not can range from their attributes, their neighborhood information (e.g., neighbor nodes attributes, connected edges' attributes, induced subgraph topology), etc. (Zhang and Tong, 2016; Yan et al., 2021b; Zhou et al., 2021). When aligning two graphs in the real world, the inevitable problem is that the input graphs are evolving in terms of features and topological structures. To this end, Yan et al. (2021a) combine two graphs into one graph, and then propose the GNN-based fast computation graph alignment method instead of re-training the GNN from scratch for each update of the combined graph. Specifically, authors want to encode the topology-invariant node embedding by training a GNN model, then fine-tune this trained GNN model with updated local changes (e.g., added nodes and edges, updated node input features). Thus, to weaken the coupling between the graph topology (e.g., adjacency matrix **A**) and the GNN parameter matrix [e.g., **W**^(*k*)^ at the *k*-th layer], authors select GCN (Kipf and Welling, 2017) as the backbone and change its information aggregation schema by introducing a topology-invariant mask gate $M^{\left(k\right)}$ and a highway gate $T^{\left(k\right)}$ as follows.


(5) $$\begin{array}{l} \;H^{\left(k\right)}=\sigma (\hat{A}{ℳ}^{\left(k-1\right)}(H^{\left(k-1\right)})W^{\left(k-1\right)})\\ H^{\left(k\right)}={𝒯}^{\left(k-1\right)}(H^{\left(k-1\right)})⊙H^{\left(k\right)}+(1-{𝒯}^{\left(k-1\right)}(H^{\left(k-1\right)}))\\ \;⊙H^{\left(k-1\right)} \end{array}$$

where ⊙ denotes Hadamard product, topology-invariant mask gate $M^{\left(k-1\right)}\left({\text{H}}^{\left(k-1\right)}\right)$ equals to ${\text{H}}^{\left(k-1\right)}⊙\sigma \left({\text{W}}_{m}^{\left(k-1\right)}\right)$, highway gate $T^{\left(k-1\right)}\left({\text{H}}^{\left(k-1\right)}\right)$ is expressed as $\sigma \left(M^{\left(k-1\right)}\left({\text{H}}^{\left(k-1\right)}\right){\text{W}}_{h}^{\left(k-1\right)}\right)$, and ${\text{W}}_{m}^{\left(k-1\right)}$ and ${\text{W}}_{h}^{\left(k-1\right)}$ are learnable parameters of $M^{\left(k-1\right)}$ and $T^{\left(k-1\right)}$. The training loss function depends on whether the embedding vectors of two paired nodes (i.e., positive samples) are close, and whether the embedding vectors of two not paired nodes (i.e., negative samples) are far away. With this trained GNN model, future updates can be regarded as additional training samples to fine-tune the model.

#### 3.1.2. Artificial dynamics in graph mining

**Graph secure generation or graph anonymization**. Graph generation is the task that models the given graphs' distribution and then generates many more meaningful graphs, which could contribute to various applications (Bonifati et al., 2020). However, approximating the observed graph distributions as much as possible will induce a privacy-leak risk in the generated graphs. For example, a node's identity is highly likely to be exposed in the generated social network if its connections are mostly preserved, which means a degree-based node attacker will easily detect a vulnerability in the generated graph with some background knowledge (Wu et al., 2010). Therefore, graph secure generation or graph anonymization is significant to social security (Fu et al., 2022c).

To protect privacy during the graph generation, artificial dynamics can help by introducing the perturbations during the modeling (or learning) of graph distributions. However, adding this kind of artificial dynamics to protect graph privacy still serves for the static graph generation. How to add dynamics to evolving graphs to protect privacy is still an opening question.

For privacy-preserving static graph generation, current solutions can be roughly classified into two types. First, the artificial dynamics is directly performed on the observed topology to generate new graph data, to name a few,

- Randomize the adjacency by iteratively switching existing edges {(*t, w*) and (*u, v*)} with {(*t, v*) and (*u, w*)} (if (*t, v*) and (*u, w*) do not exist in the original graph *G*), under the eigendecomposition preservation (Ying and Wu, 2008).
- Inject the connection uncertainty by iteratively copying each existing edge from original graph *G* to a initial null graph *G*′ with a certain probability, guaranteeing the degree distribution of *G*′ is unchanged compared with *G* (Nguyen et al., 2015).
- Permute the connection distribution by proportionally flipping the edges (existing to non-existing and vice versa), maintaining the edge-level differential privacy (edge-DP) for the graph structural preservation (Qin et al., 2017).

Second, following the synergy of deep learning and differential privacy (Abadi et al., 2016), another way to add artificial dynamics is targeting the gradient of deep graph learning models. To be specific, a deep graph generative model is recently proposed under privacy constraints, i.e., in Yang et al. (2021), privacy protection mechanism is executed during the gradient descent phase of the generation learning process, by adding Gaussian noise to the gradient.

In terms of how to design appropriate artificial dynamics for the evolving graph secure generation, it is still a challenging problem because of maintaining privacy guarantee and utility preservation simultaneously. Here we would like to share our thoughts that the next-generation techniques should address the following challenges, at least.

- Unlike static graphs, what kind of natural dynamic information is sensitive in evolving graphs and should be hidden in the generated graph to protect entities' privacy is not clear.
- After the sensitive information is determined, the protection mechanism in the evolving environment is not yet available, e.g., dealing with changing topology and features.
- When the corresponding protection mechanism is designed, it can still be challenging to maintain the generation utility at the same time with privacy constraints.

#### 3.1.3. Natural + artificial dynamics in graph mining

As mentioned in the above subsection, not only for the graph secure generation, adding artificial dynamics to evolving graphs is still nascent in many graph mining tasks, and exists many research opportunities. Here, we introduce a recent work that adds artificial dynamics to the time-evolving graph partitioning to improve computation efficiency.

**Figure 2:**
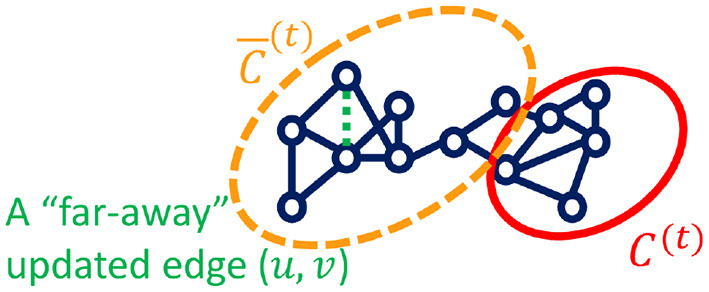
Local cluster *C*^(*t*)^ and a “far-away” edge to be filtered at time *t*.

**Node clustering or graph partitioning**. In the node clustering family, local clustering methods target a specific seed node (or nodes) and obtain the clustering by searching the neighborhood instead of the entire graph. In this paper (Fu et al., 2020e), authors propose the motif-preserving local clustering method on temporal graphs called L-MEGA, which approximately tracks the local cluster position at each timestamp instead of solving it from scratch. To make L-MEGA more efficient, one speedup technique is proposed in Fu et al. (2020e) to filter the new arrival edges instead of letting them go into the tracking process and save them for future timestamps, if the new arrival edges are “far-away” from the current local cluster and do not affect the local structure as shown in Figure 2. By doing which, the tracking time complexity can be saved. In order to investigate whether a new arrival edge can be filtered, the authors identify the “far-away” edges by analyzing its incident nodes in terms of the probability mass in the personal PageRank vector and the shortest path to the local cluster.

### 3.2. Dynamics in graph representations

In this section, we mainly discuss graph embedding (i.e., graph representation learning) as one instance of graph representations, and introduce related works about how natural dynamics and artificial dynamics are involved in boosting the performance of graph representation learning.^4^

#### 3.2.1. Natural dynamics in graph representations

In the early stage, inspired by DeepWalk (Perozzi et al., 2014), LINE (Tang et al., 2015), and node2vec (Grover and Leskovec, 2016), the graph embedding methods for temporal graphs are proposed, like CDTNE (Nguyen et al., 2018), DyGEM (Goyal et al., 2018), DynamicTriad (Goyal et al., 2018), HTNE (Zuo et al., 2018), FiGTNE (Liu et al., 2020), and tdGraphEmbd (Beladev et al., 2020). They vary in different ways to deal with time information. For example, FiGTNE (Liu et al., 2020) utilizes the temporal random walk to sample time-adjacent nodes. In this sampled sequence, the embedding is regularized such that previous nodes should reflect the current node.

Recently, inspired by GNNs stacking layers to aggregate multi-hop neighbor information to get node embedding vectors, temporal graph neural networks (TGNNs) are proposed to consider time information when doing the information aggregation, like EvolveGCN (Pareja et al., 2020), TGAT (Xu et al., 2020), and many others. In some works, they are also called spatial-temporal graph neural networks (STGNNs) because the spatial information comes from the input graph topological structure (Wu et al., 2021). In this paper, we use the term temporal graph neural networks, i.e., TGNNs, and the detailed related works for TGNNs are introduced in Section 3.3.1, i.e., *Natural Dynamics in Graph Neural Networks*.

**Multiple evolution patterns in representation learning**. As discussed earlier, in the real world, an evolving graph may have multiple evolution patterns (Fu and He, 2021a). Therefore, how to integrate multiple evolution patterns jointly during the representation learning process is still a nascent problem.

**Figure 3:**
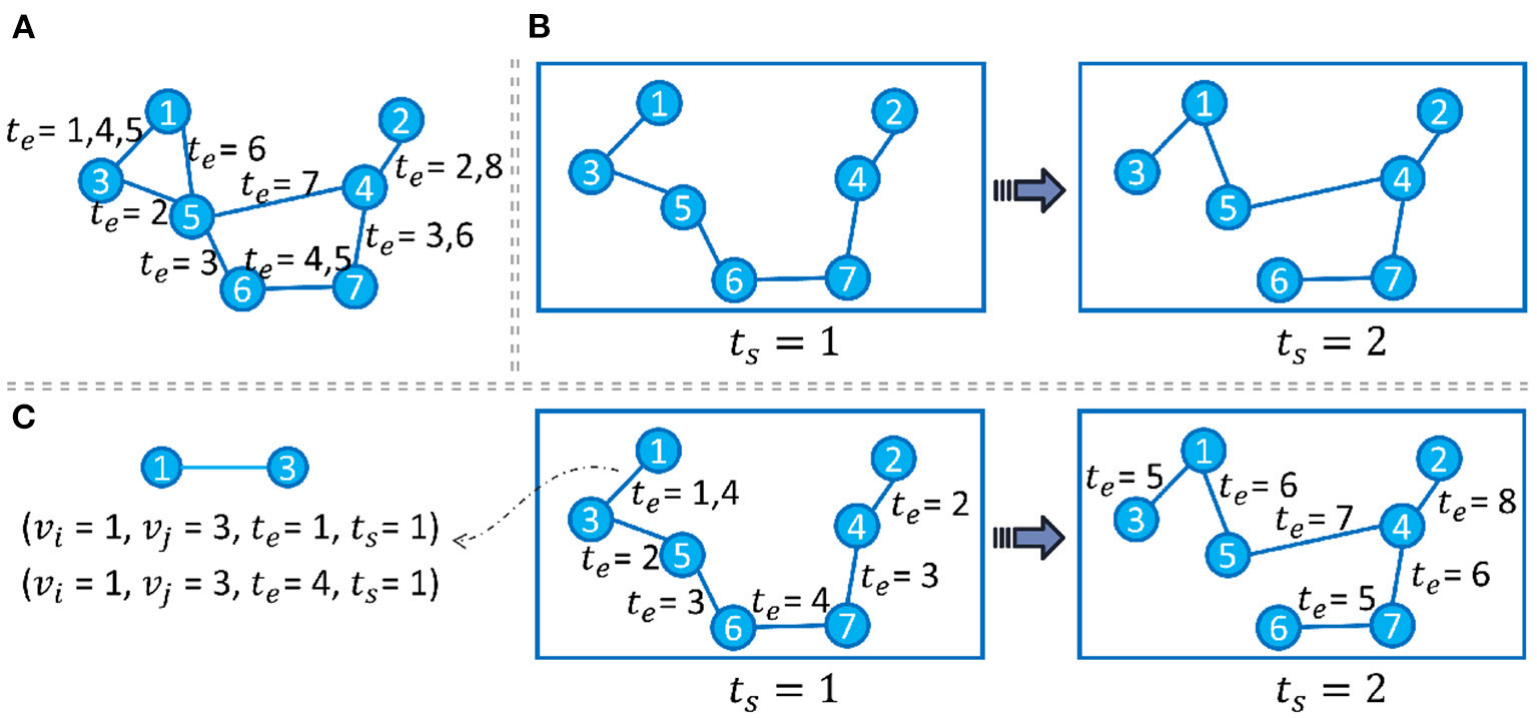
Panel **(A)** shows a streaming graph with only edge timestamps *t*_*e*_. Panel **(B)** shows a snapshot-modeled graph with only snapshot timestamps *t*_*s*_, where each *t*_*s*_ elapses every 4 *t*_*e*_. Panel **(C)** shows our multi-time evolution modeling with edge timestamps *t*_*s*_ and snapshot timestamps *t*_*e*_.

Generally speaking, if we model each evolution pattern as a different view of the input graph, then VANE (Fu et al., 2020d) could get the node embedding that is suitable for each observed view. Specifically, Temp-GFSM (Fu et al., 2022b) is proposed, which deliberately targets the streaming pattern for rapid node/edge-level evolution and the snapshot pattern for episodic and slowly-changing evolution, as shown in Figure 3. In Temp-GFSM, a multi-time attention mechanism is introduced with the support of the time kernel function to get the node-level, snapshot-level, and graph-level embeddings across different evolution patterns.

#### 3.2.2. Artificial dynamics in graph representations

**Pre-training for representation learning with masked graph signals**. Generally speaking, training graph representation learning models (e.g., GNNs) is usually executed in the (semi-)supervised setting that requires a considerable amount of labeled data, especially when the input graphs are large. However, in some domains (e.g., healthcare, Choi et al., 2017), collecting high-quality labeled graph data is usually time-consuming and costly. Therefore, recent advances have focused on the GNN pre-training (Hu W. et al., 2020; Hu Z. et al., 2020; Qiu et al., 2020; Li et al., 2021; Xu et al., 2021; Zhou et al., 2022), which pre-trains GNN models on the source domain(s) via proxy graph signals and then transfers pre-trained GNNs to the target domain. One common way of realizing proxy graph signal learning is to mask the input graphs in the unit of graph signals and train the GNNs such that they can predict the masked signals from the unmasked part. The masked signals range from masked node/edge/subgraph attributes and masked topology (e.g., nodes and edges) (Hu W. et al., 2020; Hu Z. et al., 2020).

The quality of pre-trained GNNs can largely rely on (1) the relevance between the source domain(s) and the target domain and (2) the selection of masked graph signals, which may cause the negative transfer (Rosenstein et al., 2005) if (1) the source domain distribution diverges from the target domain distribution (i.e., cross-graph heterogeneity) or masked graph signals contradict each other (i.e., graph-signal heterogeneity) (Zhou et al., 2022). Inspired by that, Zhou et al. (2022) propose the MentorGNN to realize the domain-adaptive graph pre-training. To address the cross-graph heterogeneity, MentorGNN utilizes the multi-scale encoder–decoder architecture, such that knowledge transfer can be done in a coarser resolution (i.e., transfer the encoded source domain knowledge and decode it in the target domain) instead of being directly translated. The intuition behind this is that it is more common for different domain graphs to share high-level knowledge than very detailed knowledge. To address the graph-signal heterogeneity, MentorGNN dynamically re-weighting the importance of different kinds of masked graph signals via the curriculum learning framework in terms of the target domain performance.

#### 3.2.3. Natural + artificial dynamics in graph representations

**Inserting masks to preserve evolution patterns during temporal graph representation learning**. Compared with baseline methods designed for static graph representation learning, considering the temporal information is more challenging and requires more consideration, like how to capture the evolution patterns of input graphs. In DySAT (Sankar et al., 2020), besides using structural attention like GAT (Velickovic et al., 2018) in each observed snapshot, authors design the temporal self-attention to get the node representation sequence from the first timestamp to the last timestamp, i.e., ${\text{z}}_{v}=\left\{{\text{z}}_{v}^{\left(1\right)},{\text{z}}_{v}^{\left(2\right)},\ldots ,{\text{z}}_{v}^{\left(T\right)}\right\}$, for node *v* at each observed timestamp. To preserve the evolution patterns when encoding **z**_*v*_, authors design the mask matrix **M** as follows.


(6) $$\begin{array}{l} Z_{v}=B_{v}(X_{v}W_{v}),\;B_{v}(i,j)=\frac{exp(e_{v}^{ij})}{\sum_{k=1}^{T}exp(e_{v}^{ik})}\\ e_{v}^{ij}=(\frac{(X_{v}W_{q}){\left(X_{v}W_{k}\right)}_{ij}^{⊤}}{\sqrt{F}}+M(i,j)),\;i,j\in \{1,\ldots ,T\} \end{array}$$

where matrices ${\text{W}}_{q}\in {\mathbb{R}}^{D\times F}$, ${\text{W}}_{k}\in {\mathbb{R}}^{D\times F}$, and ${\text{W}}_{v}\in {\mathbb{R}}^{D\times F}$ are query, key, value matrices in the standard self-attention mechanism (Vaswani et al., 2017). ${\text{X}}_{v}\in {\mathbb{R}}^{T\times D}$ is the node feature of node *v* across all *T* timestamps, and ${\text{Z}}_{v}\in {\mathbb{R}}^{T\times F}$ is the output time-aware representation matrix of node *v*. And $e_{v}^{ij}$ is the attention weight of timestamp *i* to timestamp *j* for node *v*, which is obtained through the mask matrix **M** ∈ ℝ^*T*×*T*^.


(7) $$M(i,j)=\{\begin{array}{ll} 0, & i\leq j\\ -\infty , & \text{otherwise} \end{array}$$

The introduction of **M** preserves the evolution pattern in an auto-regressive manner. To be specific, when **M**(*i, j*) = −∞, the softmax attention weight **B**_*v*_(*i, j*) = 0, which turns off the attention weight from timestamp *i* to timestamp *j*.

### 3.3. Dynamics in graph neural networks

In this section, we focus on a specific kind of graph representation learning tool, graph neural network (GNN), and see how natural dynamics and artificial dynamics work in GNNs.^5^

#### 3.3.1. Natural dynamics in graph neural networks

**Temporal graph neural networks (TGNNs)**. For TGNNs, the general principle is that the input graphs are evolving, e.g., the graph structure or node attributes are dependent on time. Since TGNNs take the graphs as input and the topological information is also called spatial information in some applications like traffic modeling (Li et al., 2018; Yu B. et al., 2018), TGNNs are also called spatial-temporal graph neural networks (STGNNs or ST-GNNs) in some works (Wu et al., 2021). Here, we use the term TGNNs. How to deal with time information appropriately during the vanilla GNNs' information aggregation process is the key idea for TGNNs. Different works propose different manners, not limited to the following list.

- CNN-based TGNNs: In Yan et al. (2018) and Yu B. et al. (2018), authors apply the convolutional operations from convolutional neural networks (CNNs) on graphs' evolving features to capture time-aware node hidden representations.
- RNN-based TGNNs: In Li et al. (2018), Hajiramezanali et al. (2019), and Pareja et al. (2020), authors inserts the recurrent units (from various RNNs such like LSTM and GRU) into GNNs to preserve the temporal dependency during the GNNs' representation learning process.
- Time Attention-based TGNNs: In Sankar et al. (2020), authors propose using the self-attention mechanism on time features to learn the temporal correlations along with node representations.
- Time Point Process-based TGNNs: In Trivedi et al. (2019), authors utilize Time Point Process to capture the interleaved dynamics and get time features.
- Time Kernel-based TGNNS: In Xu et al. (2020), authors use Time Kernel to project time to a differential domain for the time representation vectors.

Let's take TGAT (Xu et al., 2020) as an instance of TGNNs, to illustrate the mechanism of encoding the temporal information into the node representations. TGAT uses the Time Kernel function 𝕂 to project every observed time interval of node connections into a continuous differentiable functional domain, i.e., 𝕂:[*t*−Δ*t, t*] → ℝ^*d*^, in order to represent the time feature during the information aggregation mechanism of GNNs. Since TGAT is inspired by the self-attention mechanism (Vaswani et al., 2017), another benefit of introducing the Time Kernel is that the projected hidden representation vector can serve as the positional encoding in the self-attention mechanism. Time Kernel 𝕂 can be realized by different specific functions (Xu D. et al., 2019). For example, in TGAT (Xu et al., 2020),


(8) $$\begin{array}{l} \text{𝕂}\left(t_{e}-\text{Δ}t,t_{e}\right)=\text{Ψ}\left(t_{e}-\left(t_{e}-\text{Δ}t\right)\right)=\text{Ψ}\left(\text{Δ}t\right) \end{array}$$

and


(9) $$\begin{array}{l} \text{Ψ}\left(\text{Δ}t\right)=\sqrt{\frac{1}{d}}\left[\cos\omega_{1}\left(\text{Δ}t\right),\cos\omega_{2}\left(\text{Δ}t\right),\ldots ,\cos\omega_{d}\left(\text{Δ}t\right)\right] \end{array}$$

where Δ*t* = *t*_*e*_−(*t*_*e*_−Δ*t*) denotes the input time interval, and {ω_1_, …, ω_*d*_} are learnable parameters.

With the above time encoding, TGAT can learn node representation ${\text{h}}_{v}^{\left(t\right)}$ for node *v* at time *t* through a self-attention-like mechanism. Especially, TGAT sets node *v* as the query node to query and aggregate attention weights from its one-hop time-aware neighbors, $N_{v}^{\left(t\right)}$, to get ${\text{h}}_{v}^{\left(t\right)}$. In $N_{v}^{\left(t\right)}$, for each neighbor node *v*′, its node feature is the combination of the original input feature with the time kernel feature, i.e, $\left[{\text{x}}_{v^{\prime }}||𝕂\left(t^{\prime },t\right)\right]\in {\mathbb{R}}^{\left(m+d\right)}$, where ${\text{x}}_{v^{\prime }}\in {\mathbb{R}}^{m}$ is the original input feature of node *v*′, *K*(*t*′, *t*)∈ℝ^*d*^ is the encoded temporal feature, and *t*′ is the time when node *v*′ and *v* connects.

#### 3.3.2. Artificial dynamics in graph neural networks

**Graph augmentation for GNNs**. One straightforward example to show artificial dynamics in GNNs is the graph augmentation designed for GNNs. In general, drop operations can also be considered a kind of augmentation operation (Rong et al., 2020). Because dropping parts of the input graph can make a new input graph, such that the volume and diversity of input graphs increase. In this viewpoint, at least, graph augmentation for GNNs can be categorized into three items.

- Only drop operation: In Rong et al. (2020), authors propose DropEdge to drop a certain amount of edges in the input graphs before each epoch of GNN training, to alleviate the over-fitting problem of GNNs. Similar operations also include DropNode (Feng et al., 2020).
- Only add operation: In Gilmer et al. (2017), authors propose to add a master node to connect all existing nodes in the input graph, which operation could serve as a global scratch for the message passing schema and transfer long distance information, to boost the molecule graph prediction.
- Refine operation: In Jin et al. (2020), authors consider the problem setting given the input graph is not perfect (e.g., the adjacency matrix is poisoning attacked by adversarial edges). To be specific, they aim to investigate the low-rank property and feature smoothness to refine (i.e., not restricted to only adding or dropping) the original input graph and obtain the satisfied node classification accuracy.

More detailed operations like those mentioned above can be found in Ding et al. (2022), where these augmentation operations can also be further categorized into learnable actions and random actions.

**Adding residual connections among GNN layers**. When the input graph is imperfect (Xu et al., 2022) [e.g., topology and features are not consistent, features are partially missing], stacking more layers in GNNs can aggregate information from more neighbors to make the hidden representation more informative and serve various graph mining tasks (Zheng et al., 2022). However, the vanishing gradient problem hinders the neural networks from being deeper by making it hard-to-train, i.e., both the training error and test error of deeper neural networks are higher than shallow ones (He et al., 2016). The vanishing gradient problem can be illustrated as the gradients of the first few layers vanish, such that the training loss cannot be successfully propagated through deeper models. Currently, nascent deeper GNN methods (Li et al., 2019; Rong et al., 2020; Zhao and Akoglu, 2020) solve this problem by adding residual connections (i.e., ResNet, He et al., 2016) on vanilla GNNs. In a recent study (Zheng et al., 2022), authors find that ResNet ignores the non-IID property of graphs, and directly adding ResNet on deeper GNNs will cause the shading neighbors effect. This effect distorts the topology information by making faraway neighbor information more important in deeper GNNs, such that it adds noise to the hidden representation and degrades the downstream task performance.

**Figure 4:**
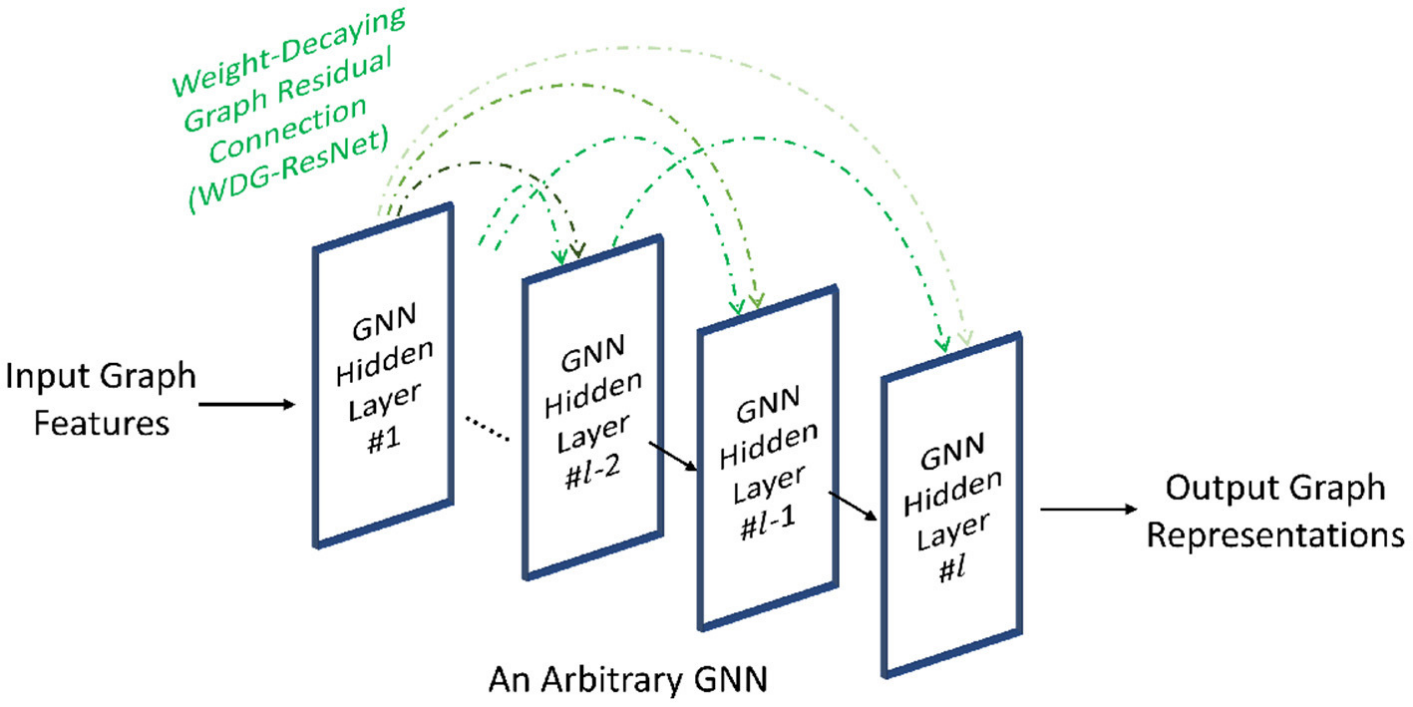
Adding weight-decaying residual connections on an arbitrary GNN architecture.

To address the shading neighbors effect, Zheng et al. (2022) design the weight-decaying graph residual connection (i.e., WDG-ResNet) deliberately for GNNs, as shown in Figure 4, which is expressed as follows.


(10) $$\begin{array}{l} {\tilde{H}}^{\left(k\right)}=\sigma (\hat{A}H^{\left(k-1\right)}W^{\left(k-1\right)}),\;\\ \text{ /*}l\text{-th layer of an arbitrary GNN},\text{ e}.\text{g}.,\text{ GCN*/}\\ H^{\left(k\right)}=sim(H^{\left(1\right)},{\tilde{H}}^{\left(k\right)})\cdot e^{-k/\lambda }\cdot {\tilde{H}}^{\left(k\right)}+H^{\left(k-2\right)},\;\\ \text{ /*residual connection*/}\\ \;=e^{cos(H^{\left(1\right)},{\tilde{H}}^{\left(k\right)})\;-\;k/\lambda }\cdot {\tilde{H}}^{\left(k\right)}+H^{\left(k-2\right)} \end{array}$$

where $cos\left({\text{H}}^{\left(1\right)},{\tilde{\text{H}}}^{\left(k\right)}\right)=\frac{1}{n}\underset{i}{\sum }\frac{{\text{H}}_{i}^{\left(1\right)}{\left({\tilde{\text{H}}}_{i}^{\left(l\right)}\right)}^{⊤}}{||{\text{H}}_{i}^{\left(1\right)}||||{\tilde{\text{H}}}_{i}^{\left(l\right)}||}$ measures the similarity between the *k*-th layer and the 1-st layer, and ${\text{H}}_{i}^{\left(1\right)}$ is the hidden representation of node *i* at the 1-st layer. The term *e*^−*l*/λ^ is the decaying factor to further adjust the similarity weight of ${\tilde{\text{H}}}^{\left(l\right)}$, where λ is a constant hyperparameter. Compared to the vanilla ResNet (He et al., 2016), the WDG-ResNet introduces the decaying factor to preserve the hierarchical information of input graphs when the GNNs go deeper to alleviate the shading neighbors effect. Moreover, the authors empirically show that the optimal decaying factor is close to the diameter of input graphs, and such heuristics reduce the search space for hyperparameter optimization.

#### 3.3.3. Natural + artificial dynamics in graph neural networks

**Augmenting temporal graphs for TGNNs**. Augmenting evolving graphs has considerable research potential but has not attracted much attention yet (Ding et al., 2022). MeTA, Wang et al. (2021a) proposes an adaptive data augmentation approach for improving temporal graph representation learning using TGNNs. The core idea is modeling the realistic noise and adding the simulated noise to the low-information area of graphs (e.g., long time and far neighbors), in order to decrease the noise uniqueness for de-overfitting and increase the generalization ability of temporal graph representation learning process, to finally help downstream tasks such as link prediction. In Wang et al. (2021a), authors propose three augmentation strategies: (1) perturbing time by adding Gaussian noise; (2) removing edges with a constant probability; (3) adding edges (i.e., sampled from the original graph) with perturbed time.

Research about augmenting temporal graphs is still in the nascent stage. And we would like to share, at least, the following research directions.

- Data-driven and learnable augmentation strategies for temporal graphs.
- Bounded augmentation solutions on temporal graphs, i.e., evolution patterns of original graphs can be preserved to some extent.
- Transferable and generalizable augmentation techniques across different temporal graphs.

## 4. Discussion and summary

In this paper, we first disentangle the graph-based research into three aspects (i.e., graph mining, graph representations, and GNNs) and then introduce the definition of natural and artificial dynamics in graphs. After that, we introduce related works in each combination between {graph mining, graph representations, and GNNs} and {natural dynamics, artificial dynamics, and natural + artificial dynamics}. In general, the topic of natural + artificial dynamics (i.e., adding artificial dynamics to evolving graphs) is still open in many graph research areas like graph mining, graph representations, and GNNs, and we list several opportunities in each corresponding subsection above. All opinions are authors' own and to the best of their knowledge. Also, due to the time limitation, many outstanding works are not discussed in this paper. We hope this paper can provide insights to relevant researchers and contribute to the graph research community.

## Author contributions

All authors listed have made a substantial, direct, and intellectual contribution to the work and approved it for publication.

## Funding

This work is supported by National Science Foundation under Award No. IIS-1947203, IIS-2117902, and IIS-2137468.

## Conflict of interest

The authors declare that the research was conducted in the absence of any commercial or financial relationships that could be construed as a potential conflict of interest.

## Publisher's note

All claims expressed in this article are solely those of the authors and do not necessarily represent those of their affiliated organizations, or those of the publisher, the editors and the reviewers. Any product that may be evaluated in this article, or claim that may be made by its manufacturer, is not guaranteed or endorsed by the publisher.

## Author disclaimer

The views and conclusions are those of the authors and should not be interpreted as representing the official policies of the funding agencies or the government.

## Footnotes

^1^Research topics like graph theory and graph database management are also very important, but we skip discussing them in this paper.

^2^As another kind of powerful tool for graph embedding, graph neural networks (GNNs) become popular and attract research attention from both the deep learning domain and graph theory domain. Here, “they” are not referring to graph neural networks. And we set up another section for introducing GNNs, otherwise Section 2.2 will be enormous and overstaffing. GNNs will be discussed separately in Section 2.3. We would like to note that GNN is a tool for realizing graph embedding as we illustrated in Figure **??**, the context separation in the paper is not standing for the tied hierarchy of graph representations and graph neural networks.

^3^The future embedding vector estimation for users and items is skipped here.

^4^Here, we select graph embedding (i.e., graph representation learning) as an instance of graph representations to introduce the corresponding natural and artificial dynamic techniques. Since GNN is a also kind of tool for graph representation learning, then in this Section 3.2, we introduce the dynamic techniques that can be applied to general graph representation learning models. In Section 3.3, for GNNs, we will introduce the dynamic techniques that are deliberately designed for GNNs, which may or may not be applied to the general graph embedding models like DeepWalk, LINE, node2vec, etc.

^5^As mentioned earlier, in this Section 3.3 we introduce the natural and artificial dynamic techniques that are deliberately designed for GNNs, which may or may not be applied to the general graph embedding models.
